# Supplementary material for: The DNA Damage Response Is Differentially Involved in HPV-Positive and HPV-Negative Radioresistant Head and Neck Squamous Cell Carcinoma
Source: Cancers (Basel). 2021 Jul 23;13(15):3717. doi: 10.3390/cancers13153717 (PMC8345136; doi:10.3390/cancers13153717)
Supplement: Supplementary file 1 [file cancers-13-03717-s001.zip › cancers-1288888-supplementary.pdf]

cancers

MDPI

Article

The DNA Damage Response is Differentially Involved in HPV-Positive and HPV-Negative Radioresistant Head and Neck Squamous Cell Carcinoma

Marieke Bamps [1,†], Rüveyda Dok [1,†] and Sandra Nuyts [1,2,*]

Supplementary Figure Legends

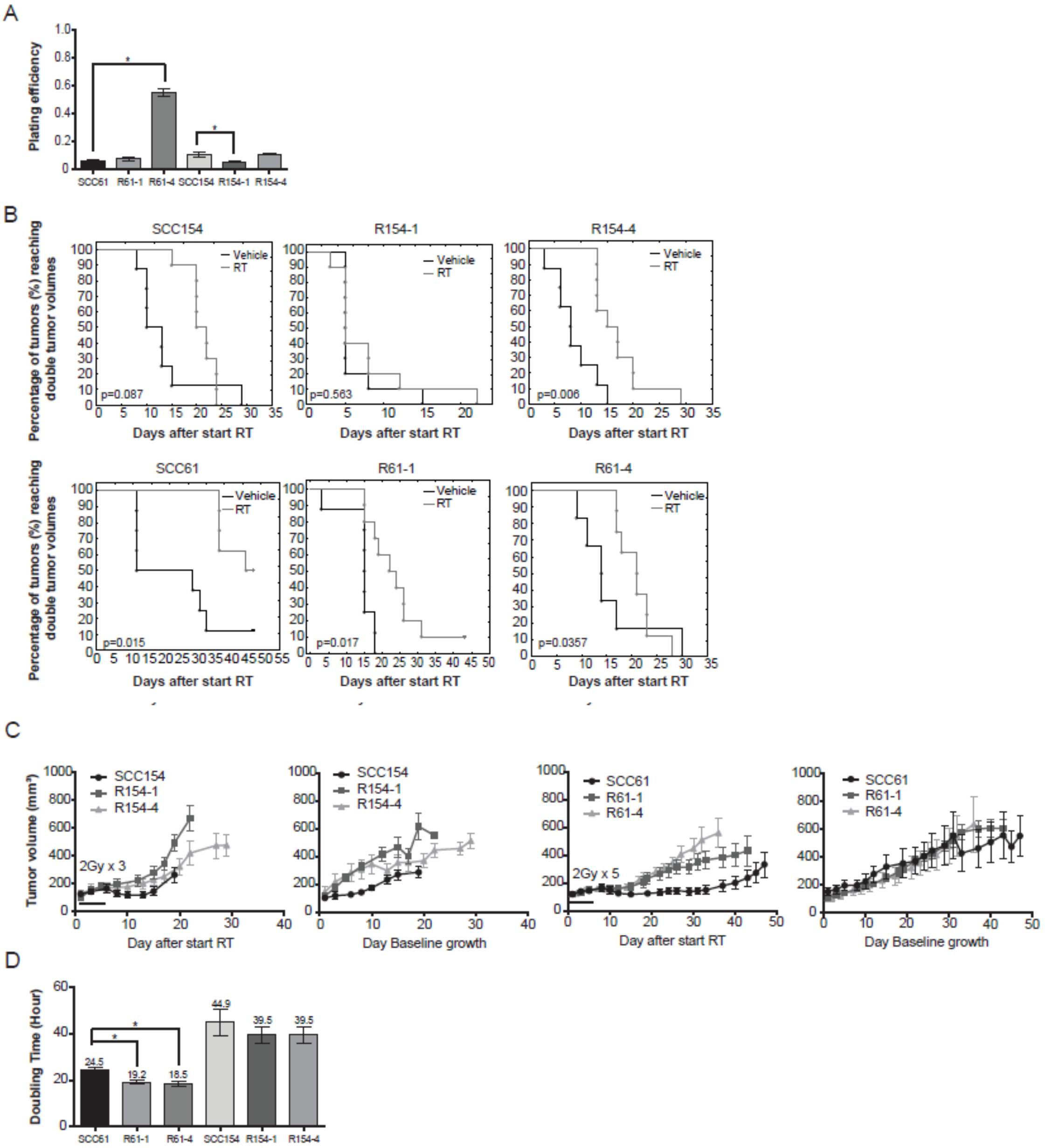

Figure S1. (A) Corresponding plating efficiencies of untreated HPV-positive and HPV2 negative parental cells and radio-resistant clones of clonogenic assays in Figure 1A. (B) Comparison of the Kaplan–Meier curves of irradiated and non-irradiated tumors of HPV-positive and HPV-negative parental and radioresistant xenografts of Figure 1B and C. Statistics

were performed by log-rank test. (C) Tumor volumes (mm^3) of irradiated and non-irradiated tumors of HPV-positive and HPV-negative xenografts of Kaplan–Meier curves of Figure 1B and C. RT was given in a fractionated manner with fractions of 2 Gy and total dose of 6 Gy and 10 Gy as indicated on the graphs. (D) Population doubling times of HPV-positive and HPV-negative radioresistant clones and parental cells. (A,D) Data are presented as the mean ± SEM for $n = 3$. * p-values < 0.05 were calculated via ANOVA with multiple comparisons test.

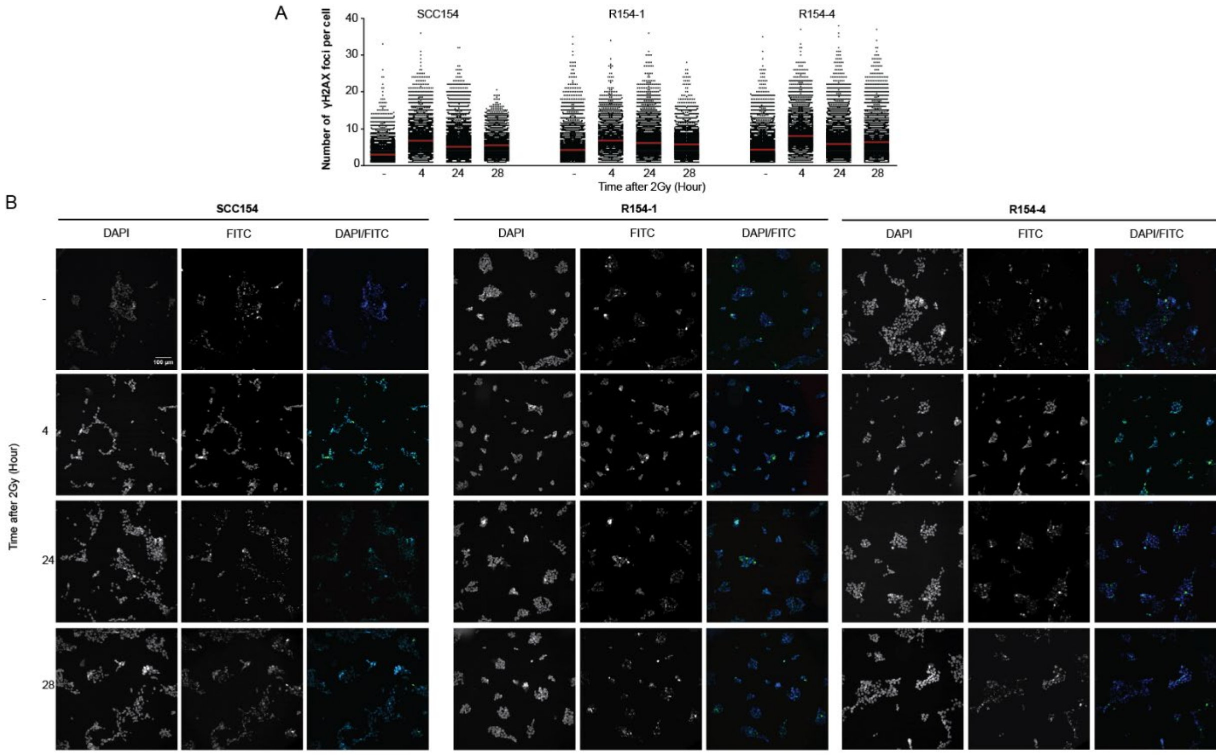

Figure S2. (A) Number of γH2AX foci per cell among all measured cells of HPV-positive parental cells and radioresistant clones in Figure 2A. γH2AX was determined at indicated time points after an RT dose of 2Gy. (B) Representative immunofluorescence images of the γH2AX foci (FITC) and corresponding nuclei (DAPI). Images were taken with IN Cell Analyzer 2000 (GE Healthcare, Chicago, IL, USA). Scale bar = 100μm.

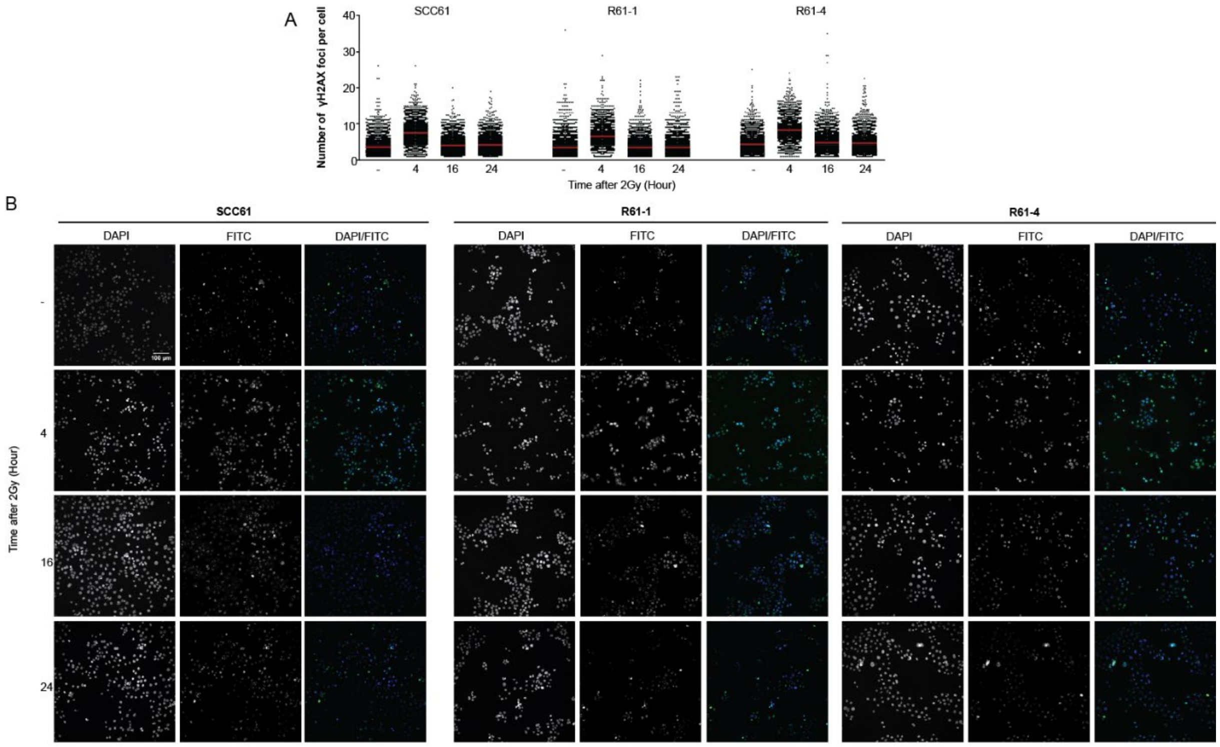

Figure S3. (A) Number of γH2AX foci per cell among all measured cells of HPV-negative parental cells and radioresistant clones in Figure 2A. γH2AX was determined at indicated time points after an RT dose of 2Gy. **(B)** Representative immunofluorescence images of the γH2AX foci (FITC) and corresponding nuclei (DAPI). Images were acquired with IN Cell Analyzer 2000 (GE Healthcare, Chicago, IL, USA). Scale bar = 100μm.

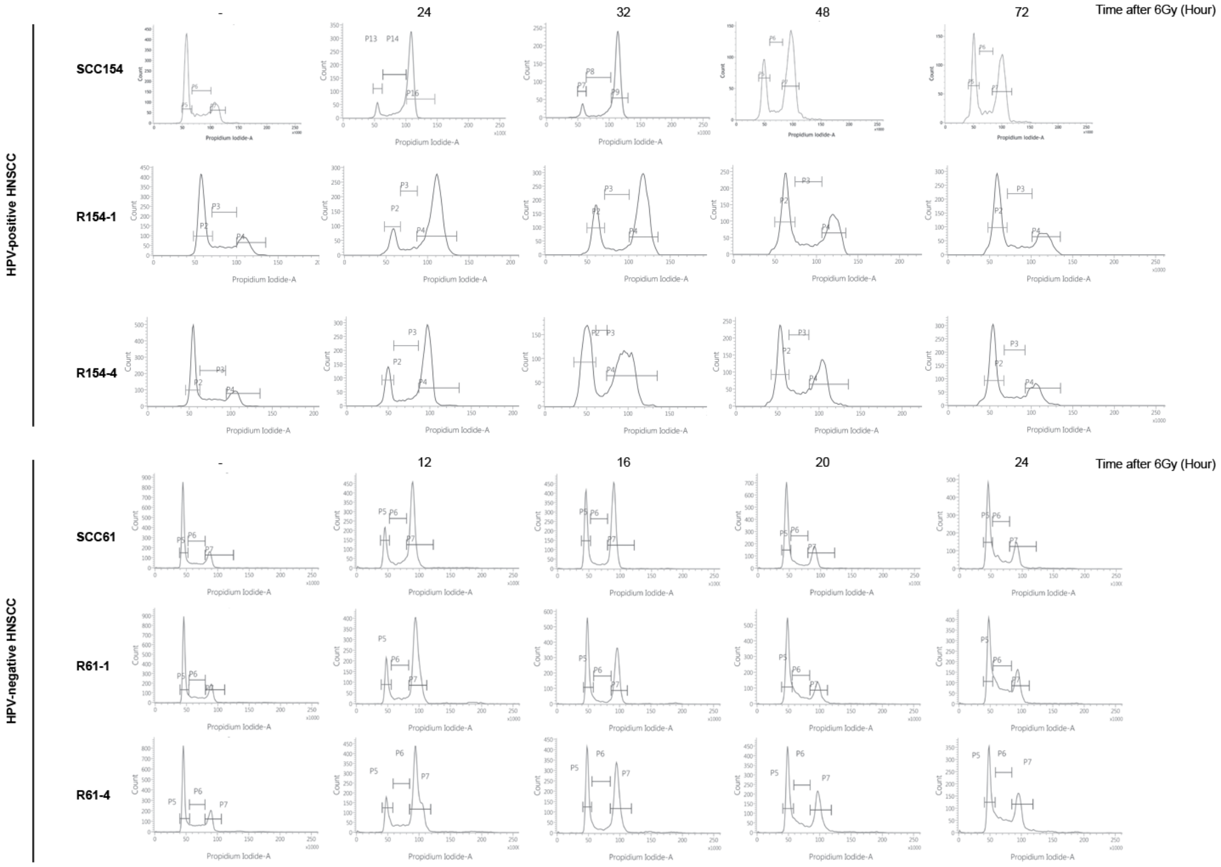

Figure S4. Representative cell cycle plots of cell cycle distribution data in Figure 2C. The cell cycle distribution of HPV-positive and HPV-negative parental cells and their radioresistant clones were assessed at unirradiated (-) conditions and at indicated time points after an RT dose of 6Gy with BD FACSVerse (Piscataway, NJ, USA).

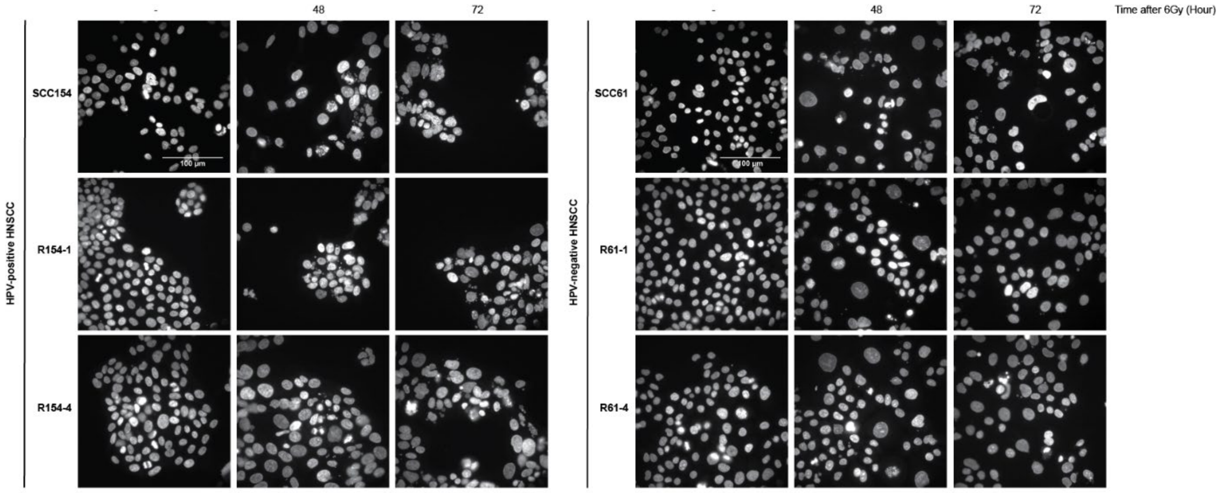

Figure S5. Representative immunofluorescence images of the micronuclei assay in Figure 2D are shown. The percentage of micronucleated cells was assessed to the total number of cells for HPV-positive and HPV-negative parental cells and their radioresistant clones at unirradiated conditions (-) and at indicated time points after an RT dose of 6Gy. Immunofluorescence images were acquired with In Cell Analyzer 2000 (GE Healthcare, Chicago, IL, USA). Scale bar = 100μm.

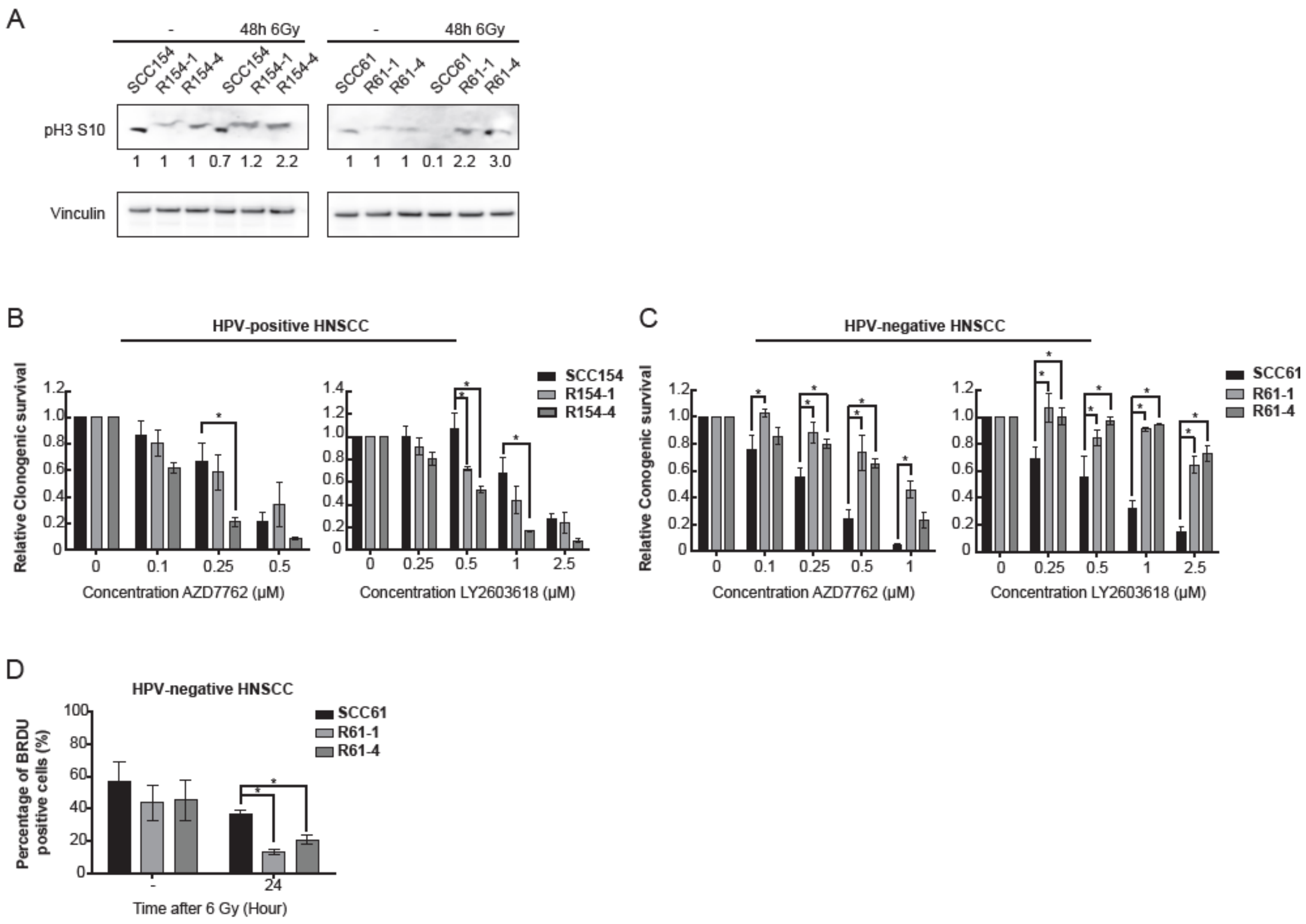

Figure S6. (A) Protein levels of pH3 S10 (17kDa) and Vinculin (124kDa) in HPV2 positive and HPV-negative parental cells and radioresistant clones in unirradiated (−) conditions and 48 h after an RT dose of 6 Gy. kDa is the molecular weight as determined by the protein standard. (B) Relative survival of HPV-positive parental cells and radioresistant clones was investigated after treatment with indicated concentrations (µM) of CHK1/2 inhibitor AZD7762 and CHK1 inhibitor LY2603618. (C) Relative survival of HPV-negative parental cells and radioresistant clones was investigated after treatment with indicated concentrations (µM) of CHK1/2 inhibitor AZD7762 and CHK1 inhibitor LY2603618. (D) Percentage of BRDU-positive cells after BRDU-incorporation for 2 hours in SCC61, R61-1 and R61-4 in unirradiated cells (−) and at 24 hours after an RT dose of 6 Gy. Data are presented as the mean ± SEM for $n = 3$. (B, C) Relative survival was determined by the means of clonogenic survival and shown as the mean ± SEM clonogenic survival fraction of drug treated conditions relative to vehicle treated control cells, $n = 3$. (B–D) * p-values < 0.05 were calculated via ANOVA with multiple comparisons test.

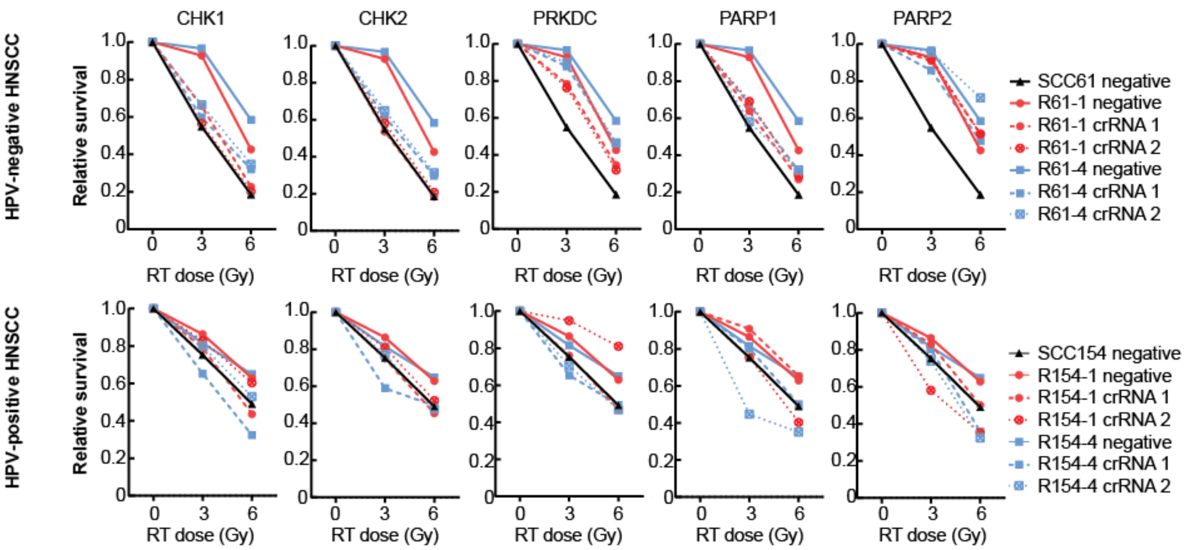

Figure S7. Relative cellular survival after indicated RT doses of HPV-positive and HPV-negative radioresistant HNSCC cells upon knockdown of indicated genes, with two CRISPR-Cas9 complexes (crRNA) for each gene. Relative cellular survival was measured by SRB assay and is shown as cellular survival after irradiation relative to non-irradiated cells, n=1.
